# *Bartonella* species bacteremia in association with adult psychosis

**DOI:** 10.3389/fpsyt.2024.1388442

**Published:** 2024-06-07

**Authors:** Shannon Delaney, Cynthia Robveille, Ricardo G. Maggi, Erin Lashnits, Emily Kingston, Chance Liedig, Lilly Murray, Brian A. Fallon, Edward B. Breitschwerdt

**Affiliations:** ^1^ Columbia University Irving Medical Center, Department of Psychiatry, New York, NY, United States; ^2^ New York State Psychiatric Institute, Department of Psychiatry, New York, NY, United States; ^3^ Intracellular Pathogens Research Laboratory, Comparative Medicine Institute, North Carolina State University, College of Veterinary Medicine, Department of Clinical Sciences, Raleigh, NC, United States; ^4^ Department of Medical Sciences, School of Veterinary Medicine, University of Wisconsin-Madison, Madison, WI, United States

**Keywords:** *Bartonella*, infection, psychosis, serology, polymerase chain reaction, neurologic diseases

## Abstract

**Introduction:**

The potential role of pathogens, particularly vector-transmitted infectious agents, as a cause of psychosis has not been intensively investigated. We have reported a potential link between *Bartonella* spp. bacteremia and neuropsychiatric symptoms, including pediatric acute onset neuropsychiatric syndrome and schizophrenia. The purpose of this study was to further assess whether *Bartonella* spp. exposure or infection are associated with psychosis.

**Methods:**

In a blinded manner, we assessed the presence of anti-*Bartonella* antibodies by indirect immunofluorescence assays (IFA), and infection by amplification of bacterial DNA from blood by quantitative polymerase chain reaction (qPCR), digital PCR (dPCR), and droplet digital PCR (ddPCR) in 116 participants. Participants were categorized into one of five groups: 1) controls unaffected by psychosis (*n* = 29); 2) prodromal participants (*n* = 16); 3) children or adolescents with psychosis (*n* = 7); 4) adults with psychosis (*n* = 44); and 5) relatives of a participant with psychosis (*n* = 20).

**Results:**

There was no significant difference in *Bartonella* spp. IFA seroreactivity between adults with psychosis and adult controls unaffected by psychosis. There was a higher proportion of adults with psychosis who had *Bartonella* spp. DNA in the bloodstream (43.2%) compared to adult controls unaffected by psychosis (14.3%, *p* = 0.021). The *Bartonella* species was determined for 18 of the 31 bacteremic participants, including infection or co-infection with *Bartonella henselae* (11/18), *Bartonella vinsonii* subsp. b*erkhoffii* (6/18), *Bartonella quintana* (2/18), *Bartonella alsatica* (1/18), and *Bartonella rochalimae* (1/18).

**Discussion:**

In conjunction with other recent research, the results of this study provide justification for a large national or international multi-center study to determine if *Bartonella* spp. bacteremia is more prevalent in adults with psychosis compared to adults unaffected by psychosis. Expanding the investigation to include a range of vector-borne and other microbial infections with potential CNS effects would enhance knowledge on the relationship between psychosis and infection.

## 1 Introduction

Psychosis constitutes a severely demoralizing illness for the patient, creates numerous emotional and medical management challenges for family members and physicians, and contributes to a substantial economic burden for society (1, 2). An increasing number of studies supports a role for chronic inflammation in various neurological conditions, including studies that have focused on patients with schizophrenia and psychosis (3–5). In a study involving 638,213 Swedish men, high erythrocyte sedimentation rate, a global indicator of inflammation, was associated with increased risk for schizophrenia and decreased risk for other non-affective psychoses in adulthood (6). Yuan and colleagues performed a systematic meta-analysis of inflammation-related factors in eight major psychiatric disorders, including schizophrenia (SCZ), bipolar disorder, autism spectrum disorder, major depression disorder, post-trauma stress disorder, sleeping disorder, obsessive-compulsive disorder and suicide (7). As their work supported the possibility of differentiating psychiatric disorders by using inflammatory biomarkers, the authors proposed a system-wide longitudinal study using strict analytical procedures to validate sensitive and specific inflammatory biomarkers associated with different types of psychosis. While identifying biomarkers of inflammation helps to clarify a potential mechanism of disease, identifying unrecognized perpetuating agents of inflammation may prove to be a more effective strategy for generating patient specific interventions in the future.

*Bartonella* spp. are emerging, potentially zoonotic pathogens that are most often transmitted by arthropod vectors or animal bites and scratches (8–12). A substantial number of animal species have co-evolved with a specific *Bartonella* sp. (now more than 45 named species), for which an animals’ blood serves as a reservoir for blood sucking arthropods. After human beings become incidentally infected, symptoms most often consist of acute onset fever, myalgia, headache and potentially lymphadenopathy (13, 14). Although the acute infection can vary in severity, most people experience a mild to moderate flu-like illness that is most often self-limiting. With the advent of more sensitive diagnostic methods, it is now recognized that some infected individuals develop a longstanding blood borne infection, accompanied by a spectrum of chronic, often non-specific symptoms primarily involving the cardiovascular (endocarditis and myocarditis), neurological (neuropathy, seizures, encephalitis, and other symptoms) and rheumatological (myalgia, fatigue, joint pain) systems (15–19). Pediatric acute onset neuropsychiatric syndrome involves new onset complex psychiatric symptoms emerging in the context of an infectious trigger; a case report has identified *Bartonella* as a potential contributing factor, with symptom resolution by using antimicrobial treatments (20). Considering the increasingly large number of *Bartonella* species, the environmental diversity of mammalian reservoir hosts, and the range of competent and suspected vectors for *Bartonella* spp. transmission, it is increasingly obvious that human exposures to this genus of bacteria are more frequent and ubiquitous than formerly suspected (11, 21–26).

Previously, the authors (Delaney S, Fallon B) investigated the potential role of inflammation in children, adolescents, and young adults with psychosis (27). They found significantly elevated C-reactive protein (CRP) levels and interleukin 6 (IL-6) in the psychosis group compared to the controls unaffected by psychosis (27). In addition, IL-6 levels correlated positively with anti-lipopolysaccharide (LPS) IgA antibodies in the psychosis group, and negatively with vitamin D. At the same time, the corresponding author and his collaborators tested people with schizophrenia or schizoaffective disorder (SCZ/SAD) for *Bartonella* infection by droplet digital PCR (ddPCR). As the study was halted due to the SARS CoV2 pandemic, the authors elected to unblind and publish the findings as a pilot study that found people with SCZ/SAD were significantly more likely than healthy volunteers to have *Bartonella* spp. DNA in their blood (28). The goal of the current study was to further assess whether *Bartonella* spp. exposure or infection are associated with psychosis. Our primary hypothesis was that detection of *Bartonella* species DNA would be significantly associated with psychotic symptoms.

## 2 Materials and methods

### 2.1 Study participants

One hundred and sixteen participants were included in this study. All participants (including children) and parents for those under age 18 signed a consent. The protocol for sample collection (#7029) was approved by the New York State Psychiatric Institute Institutional Review Board. Blood and serum specimens stored at -80°C, including individuals from a previously published cohort (27), were used for molecular and serological testing, respectively. All samples were de-identified and shipped overnight express to the Intracellular Pathogens Research Laboratory, North Carolina State University (NCSU). Investigators and research technicians at NCSU were blinded to all participant categorizations. Results obtained previously using inflammatory markers (CRP, IL-6), anti-LPS antibodies (IgM, IgG, IgA) and vitamins (D, B12, folate) were reanalyzed. Some biomarker values were not available for all participants.

Participants were classified into one of five groups: 1) controls unaffected by psychosis (ages 11–33, *n* = 29); 2) prodromal participants with psychosis (ages 16–30, *n* = 16); 3) children or adolescents with psychosis (ages 8–16, *n* = 7); 4) adults with psychosis (ages 18–37, *n* = 44); and 5) relatives of a participant with psychosis (ages 21–67, *n* = 20). Relatives were composed of parents (*n* = 15), siblings (*n* = 4), and an aunt (*n* = 1). Participants with psychosis were recruited from the community and inclusion criteria included those between the ages of 8 and 35 with a psychiatrist verified diagnosis on the MINI Neuropsychiatric structured diagnostic interview, including a positive diagnosis of psychosis (current or lifetime) or a mood disorder with psychotic symptoms. Prodromal participants were recruited solely through the prodromal clinic, the Center for Prevention and Evaluation (COPE), and met criteria for the Attenuated Positive Symptom Syndrome (APSS) (29). Controls unaffected by psychosis were recruited through Columbia’s clinical research website and denied having a history of psychotic symptoms or of autoimmune conditions. The latter were excluded from the original study (27), due to potential increased inflammatory markers. Participants mostly lived in the greater New York City area; their risk of vector exposure was not assessed.

### 2.2. Laboratory analyses

**Figure 1:**
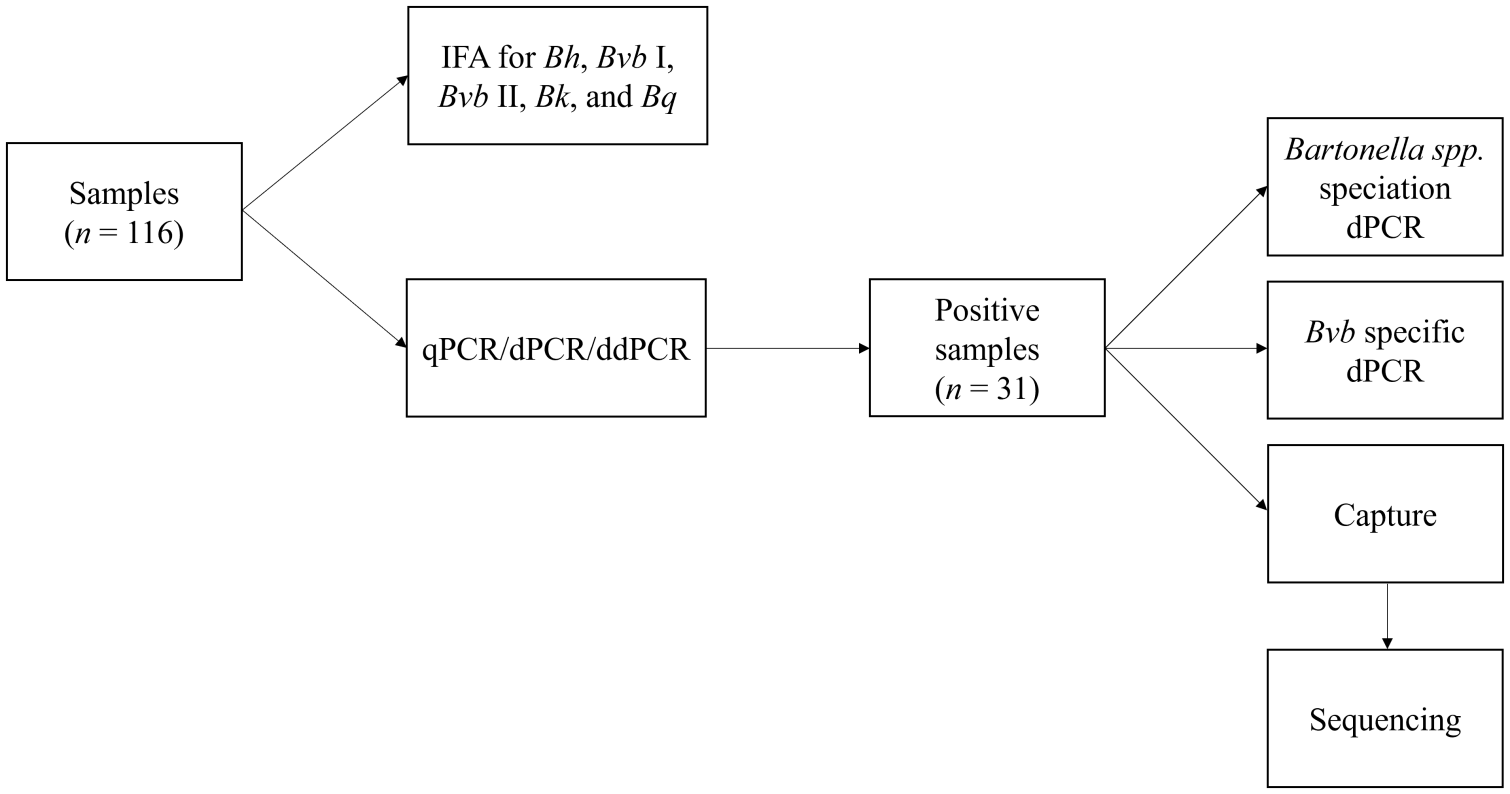
Summary of the *Bartonella* serological and molecular testing approaches used in this study. IFA, indirect fluorescent antibody; *Bh*, *Bartonella henselae*; *Bvb* I, *Bartonella vinsonii* subsp. *berkhoffii* genotype I; *Bvb* II, *Bartonella vinsonii* subsp. *berkhoffii* genotype II; *Bk*, *Bartonella koehlerae*; *Bq*, *Bartonella quintana*; qPCR, quantitative polymerase chain reaction; dPCR, digital PCR; ddPCR, droplet digital PCR.

The testing approaches used in this study are depicted in **Figure 1**.

#### 2.2.1 Serological studies

As described previously (30, 31), each participant was tested using five indirect fluorescent antibody (IFA) assays, each representing a unique *Bartonella* species or genotype. *Bartonella vinsonii* subsp. *berkhoffii* (genotypes I and II), *B. henselae* (strain San Antonio 2), *B. koehlerae*, and *B. quintana* IgG antibodies were determined using DH82 cell culture-grown bacteria as antigens and following standard IFA techniques with fluorescein conjugated goat anti-human IgG. A sample was considered *Bartonella* spp. seroreactive if an IFA titer of ≥1:64 was obtained for any one or more antigens.

#### 2.2.2 Molecular studies

Following DNA extraction from each whole blood sample, amplification of the human hydroxymethylbilane synthase gene was used as housekeeping reference gene. The *Bartonella* spp. intergenic spacer 16S-23S rRNA region was targeted by quantitative PCR (qPCR, CFXOpus thermocycler, Bio-Rad, Hercules, CA), digital PCR (dPCR, QIAcuity nanoplate-based digital PCR system, Qiagen, Carlsbad, CA), and droplet digital PCR (ddPCR, QX200 Droplet Digital PCR, Bio-Rad, Hercules, CA) using primers and probes as previously described (28, 32–34). A sample was considered PCR positive if any one or more of the qPCR, dPCR or ddPCR testing modalities generated a positive result.

Attempts to identify *Bartonella* species using PCR+ samples were performed by qPCR and dPCR using either species specific probes (with minor modifications) as previously described (35), or by biotin-streptavidin DNA amplicon capture with qPCR re-amplification, followed by DNA sequence species comparisons. In addition, DNA amplification specifically targeting the *Bartonella vinsoni* subsp. *berkhoffii* intergenic spacer region was also performed using *B. vinsoni* subsp. *berkhoffii* specific primers and probes (36).

### 2.3 Statistical analyses

Summary statistics for demographics and *Bartonella* test results were calculated for each group. For statistical analysis of the primary outcome, only unrelated adults (18 years of age or older) between the group with psychosis (*n* = 44) and the control group (group unaffected by psychosis; *n* = 28) were analyzed. Thus, one child, an 11 year-old female, in the control group was excluded in the statistical analysis. Descriptive results only are presented for prodromal participants, children or adolescents with psychosis, and relatives.

For continuous variables (age and body mass index), the case and control groups were compared using the Wilcoxon rank sum test for nonparametric data. For categorical variables (gender, *Bartonella* serology result, *Bartonella* PCR result), adults with psychosis and controls were compared using chi-squared test (or Fisher exact test for small group sizes). To determine agreement between IFA and PCR, the kappa statistic was calculated (37).

Associations between biomarkers (CRP, IL-6, serum anti-LPS IgM/IgG/IgA, vitamin D, folate, and vitamin B12) and seroreactivity or PCR status were determined using Wilcoxon rank sum test for nonparametric data. This test was also used to assess associations between biomarkers and adults with psychosis or controls. Since these biomarker comparisons were being done to generate novel hypotheses, correction for multiple comparisons was not performed to decrease the risk of alpha error in this small sample size.

Statistical significance was set at *p* ≤ 0.05. All statistical analyses were performed in R v. 4.3.2 (R Core Team 2023) (38).

## 3 Results

### 3.1 Demographic analyses

Demographic information for all groups is summarized in **Table 1**. There were 44 adults with psychosis and 28 controls included in the statistical analysis. There was no significant difference in age (*p* = 0.982) or body mass index (*p* = 0.291) between the two groups. Gender data was available for only 39 participants with psychosis and 19 controls; of those with data available, there was a higher proportion of male participants in the group with psychosis (25/39, 64.1%) compared to the control group (6/19, 31.6%, *p* = 0.040).

**Table 1:** Demographics by study group.

Group	Median age, years (range)	Gender female/male/unreported	Median body mass index(range)
Children control	11 (*n* = 1)	1/0/0	22.3 (*n* = 1)
Adult controls	24 (19–33) (*n* = 28)	13/6/8/^a^1	23 (19.9–43.3) (*n* = 28)
Prodromal participants	23 (16–30) (*n* = 16)	6/10/0	22.7 (16.9–33.1) (*n* = 16)
Children with psychosis	12 (8–16) (*n* = 7)	3/1/3	20.6 (17.9–25.1) (*n* = 4)
Adults with psychosis	24 (18–37) (*n* = 44)	14/25/5	25.1 (19.8–54.4) (*n* = 39)
Relatives	25 (21–67) (*n* = 19)	0/3/17	N/A

a Transgender participant (F→M).

N/A, not applicable (unavailable data).

### 3.2 Serological results

Serological results are summarized by study group in **Tables 2**, **3**. Overall, 72/116 (62.1%) participants were seroreactive to one or more of the five *Bartonella* spp. antigens by IFA testing. The difference in the proportion of seroreactive adults was not statistically significant between the control group (21/28, 75.0%) and the group with psychosis (25/44, 56.8%) (*p* = 0.189). *Bartonella vinsonii* subsp. b*erkhoffii* genotype II and *B. henselae* were the most frequently seroreactive species among the five antigens tested in the overall population (48.3% and 46.6%, respectively). Patterns of seroreactivity were highly variable among individuals, with some participants reactive to all five antigens (22/116; 19.0%) and others reactive to only one (18/116; 15.5%), two (13/116; 11.2%), three (10/116; 8.6%) or four antigens (9/116; 7.8%). Most seroreactive participants (54/72, 75.0%) were reactive to more than one *Bartonella* species/genotype. The percentage of seroreactive participants progressively decreased as antibody titers increased from 1:64 to 1:1024, the highest titer recorded to any antigen for any participant. Reciprocal *B. henselae* and *B. vinsonii subsp. berkhoffii* genotype I antibody titers ranged from 64 to 1024, whereas reciprocal *B. koehlerae*, *B. quintana* and *B. vinsonii* subsp. *berkhoffii* genotype II antibody titers ranged from 64 to 512.

**Table 2:** *Bartonella* spp. indirect fluorescent antibody results for 116 study participants.

Group	Titer	For at least one antigen	*Bh*	*Bvb* genotype I	*Bvb* genotype II	*Bk*	*Bq*
Controls (*n* = 29)	≥1:64	22	16	17	13	10	8
	≥1:128	11	9	6	8	3	3
	≥1:256	5	5	2	1	1	1
Prodromal participants (*n* = 16)	≥1:64	7	6	4	6	5	3
	≥1:128	4	3	2	3	0	0
	≥1:256	2	0	1	2	0	0
Children with psychosis (*n* = 7)	≥1:64	6	5	3	4	3	3
	≥1:128	4	2	3	2	0	0
	≥1:256	1	0	1	0	0	0
Adults with psychosis (*n* = 44)	≥1:64	25	17	14	24	11	11
	≥1:128	18	8	10	16	3	6
	≥1:256	10	7	7	5	2	1
Relatives (*n* = 20)	≥1:64	12	10	8	9	6	4
	≥1:128	7	6	5	5	4	4
	≥1:256	3	3	3	3	2	2
Total seroreactive (*n* = 116)	≥1:64	72	54	46	56	35	29
	≥1:128	44	28	26	34	10	13
	≥1:256	21	15	14	11	5	4
Total non-seroreactive (*n* = 116)	<1:64	N/A	62	70	60	81	87

*Bh, Bartonella henselae; Bk, Bartonella koehlerae; Bq, Bartonella quintana; Bvb, Bartonella vinsonii subsp. berkhoffii*; N/A, not applicable.

For each group, numerical values represent the number of seroreactive participants against each *Bartonella* species or genotype antigen tested at titers of ≥1:64 (1^st^ line, no shading), ≥1:128 (2^nd^ line, light shading), and ≥1:256 (3^rd^ line, dark shading). In the last row, numerical values represent the number of non-seroreactive participants against each *Bartonella* species or genotype antigen.

**Table 3:** *Bartonella* spp. indirect fluorescent antibody results for 116 study participants.

Group	Seroreactivity at ≥1:64					
	0 Ag	1 Ag	2 Ag	3 Ag	4 Ag	5 Ag
Controls (*n* = 29)	7	7	4	1	4	6
Prodromal participants (*n* = 16)	9	1	1	1	2	2
Children with psychosis (*n* = 7)	1	2	0	2	0	2
Adults with psychosis (*n* = 44)	19	6	5	3	3	8
Relatives (*n* = 20)	8	2	3	3	0	4
Total (%) (*n* = 116)	44 (37.9)	18 (15.5)	13 (11.2)	10 (8.6)	9 (7.8)	22 (19)

Ag, antigen.

For each group, numerical values represent the number of non-seroreactive or seroreactive participants against one or more *Bartonella* species or genotype antigen tested, at titers of ≥1:64. In the last row, the proportion of non-seroreactive or seroreactive participants compared to all participants is indicated.

### 3.3 Molecular and DNA sequencing results

Results of qPCR, dPCR and ddPCR testing are summarized by study group in **Table 4**. DNA from at least one *Bartonella* species was amplified in 31/116 (26.7%) participants, including 5 controls unaffected by psychosis (5/29; 17.2%), 3 prodromal participants (3/16; 18.8%), 19 adults with psychosis (19/44; 43.2%), and 4 relatives of participants with psychosis (4/20; 1 sibling and 3 parents; 20%). None of the children, except the one in the control group, was PCR+. The difference in the proportion of PCR+ adults was statistically significant between the control group (4/28, 14.3%) and the group with psychosis (19/44, 43.2%) (*p* = 0.021). The *Bartonella* species was determined for 18 of the 31 bacteremic participants (**Table 5**). *Bartonella henselae* (11/18, 61.1%) and *B. vinsonii* subsp. *berkhoffii* (6/18, 33.3%) were the most common species identified. There was co-infection in three adults with psychosis, involving *B. quintana* (2/18), *B. alsatica* (1/18), and *B. rochalimae* (1/18).

**Table 4:** Molecular results (qPCR, dPCR, ddPCR) for 116 participants tested for *Bartonella* infection.

Group	qPCR+	dPCR/ddPCR+	qPCR+ and dPCR/ddPCR+	Positivity with at least one method (%)
Controls (*n* = 29)	0	5	0	5 (17.2)
Prodromal participants (*n* = 16)	2	2	1	3 (18.8)
Children with psychosis (*n* = 7)	0	0	0	0
Adults with psychosis (*n* = 44)	7	14	2	19 (43.2)
Relatives (*n* = 20)	0	4	0	4 (20.0)
Total (%) (*n* = 116)	9 (7.8)	25 (21.6)	3 (2.6)	31 (26.7)

qPCR, quantitative polymerase chain reaction; dPCR, digital PCR; ddPCR, droplet digital PCR.

For each group, numerical values represent the number of participants for which *Bartonella* DNA was amplified. In the last row, the proportion of participants with *Bartonella* DNA amplification compared to all participants is indicated.

**Table 5:** *Bartonella* species determination for 31 bacteremic participants.

Group		*Bartonella* species			
		*Bh*	*Bvb* ^b^	*B. rochalimae* ^b^	Species undetermined
Controls (*n* = 5)	Child	1^a^	0	0	0
	Adults	1^a^	2	0	1^c^
Prodromal participants (*n* = 3)		1^a^	1	0	1^c^
Children with psychosis (*n* = 0)		0	0	0	0
Adults with psychosis (*n* = 19)		8 (incl. 1 co-infection with *Bq*)^d^	2 (incl. 1 co-infection with *B. alsatica*)	1 (co-infected with *Bq*)	8^e^
Relatives (*n* = 4)	Sibling	0	1	0	0
	Parents	0	0	0	3^c^

*B., Bartonella; Bh, Bartonella henselae; Bq, Bartonella quintana; Bvb, Bartonella vinsonii* subsp. *berkhoffii*; incl., included.

a Confirmed by DNA Sanger sequencing.

b Confirmed by species probe-based PCR.

c Confirmed by genus probe-based PCR.

d Confirmed by DNA Sanger sequencing (*n* = 7) or species probe-based PCR (*n* = 1).

e Confirmed by DNA Sanger sequencing (*n* = 2) or genus probe-based PCR (*n* = 6).

### 3.4 Agreement between serological and molecular results

Agreement between serological and molecular results was slight or less (kappa = -0.09, 95% CI -0.27–0.09) (**Tables 6**, **7**). This agreement was not significantly more likely than would be expected by chance alone (*p* = 0.335). There was seroreactivity to at least one antigen in 19 of 31 bacteremic participants (61.3%), and in 53 of 85 non-bacteremic participants (62.4%). There was no significant association between *B. henselae* PCR positivity and *B. henselae* seroreactivity (*p* = 0.549): 11% of participants with *B. henselae* titers less than 1:64 were PCR+ (7/62), compared to 5% of participants with *B. henselae* titers 1:64 or 1:128 (2/39) and 13% of participants with *B. henselae* titers 1:256 or above (2/15, including the child control). Of the six *B. vinsonii* subsp. *berkhoffii* PCR+ participants, five were *B. vinsonii* subsp. *berkhoffii* genotype I and/or II seroreactive (3 at 1:64 and one each at 1:128 and 1:256). The sole non-seroreactive participant was co-infected with *B. alsatica.* The participant that was PCR+ for *B. quintana* and *B. rochalimae* was seroreactive to all five antigens at titers of 1:256, whereas the participant co-infected with *B. quintana* and *B. henselae* was seronegative to all five antigens. One adult with psychosis, diagnosed with anti-NMDA (N-methyl-D-aspartate) receptor antibody encephalitis, was seroreactive to all five antigens at titers of 1:128 (*B*. *quintana*) or 1:256 (*B. henselae*, *B. vinsonii* subsp. *berkhoffii* genotype I and II, *B. koehlerae*); *Bartonella* spp. DNA was not amplified from the blood specimen.

**Table 6:** Agreement between *Bartonella* spp. indirect fluorescent antibody results and molecular results for each group.

**Group**	**IFA/PCR**			
	+/+	+/-	-/+	-/-
Controls (*n* = 29)	5	17	0	7
Prodromal participants (*n* = 16)	1	6	2	7
Children with psychosis (*n* = 7)	0	6	0	1
Adults with psychosis (*n* = 44)	10	15	9	10
Relatives (*n* = 20)	3	9	1	7
Total (%) (*n* = 116)	19 (16.4)	53 (45.7)	12 (10.3)	32 (27.6)

IFA, indirect fluorescent antibody; PCR, polymerase chain reaction.

A participant was considered seroreactive if the IFA titer was ≥1:64 for at least one antigen. In the last row, the proportion of participants with each of the four possible results compared to all participants is indicated.

**Table 7:** Seroreactivity to specific antigens by indirect fluorescent antibody assays in bacteremic participants.

	Monoinfection		Co-infection			
	*Bh* (*n* = 10)	*Bvb* (*n* = 5)	*Bh+Bq* (*n* = 1)	*Bvb+B. alsatica* (*n* = 1)	*Bq+B. rochalimae* (*n* = 1)	Species undetermined (*n* = 13)
IFA+ for the specific antigen	4	5	0	0	1	N/A
IFA+ only for the specific antigen	0	1	0	0	0	N/A
IFA- for the 5 antigens	4	0	1	1	0	5

*B., Bartonella; Bh, Bartonella henselae; Bq, Bartonella quintana; Bvb, Bartonella vinsonii* subsp. *berkhoffii*; N/A, not applicable.

For *Bartonella vinsonii *subsp. *berkhoffii* IFA, the result for both genotypes was merged.

### 3.5 Biomarkers results

There was no significant association between any individual biomarker (CRP, IL-6, serum anti-LPS IgM, IgG, or IgA, vitamin D, folate or vitamin B12) and seroreactivity in adults (data not shown). For the PCR status, only serum anti-LPS IgG was significantly higher in bacteremic adults (median: 0.1036782, range: 0.0203120–0.3348107, *n* = 25) compared to non-bacteremic adults (median: 0.0688245, range: 0.0145963–0.4288184, *n* = 49) (*p* = 0.028). There were no significant associations between any individual biomarker and participant group (adults with psychosis compared to controls) (data not shown).

## 4 Discussion

In this study, there was a higher proportion of adults with psychosis that had *Bartonella* spp. DNA in the bloodstream compared to adult controls unaffected by psychosis. This finding is consistent with the results of the pilot study by Lashnits and colleagues, in which a higher proportion of adults with SCZ/SAD had *Bartonella* spp. DNA amplified from blood (11 of 17 participants) compared to healthy controls (1 of 13 participants) (28). In the current study, a positive qPCR result was obtained for only 9 of 31 bacteremic participants; all of whom were either in the prodromal or the adult psychosis groups. *Bartonella* spp. DNA was only amplified by dPCR or ddPCR in the remaining 22 PCR+ participants, highlighting the enhanced sensitivity of these two digital PCR techniques when attempting to document (i.e., microbiologically detect) low template bacterial DNA concentrations in participant blood. Sanger sequencing, DNA capture, Taqman^®^ probe-based, and *B. vinsonii* subsp. *berkhoffii*-specific PCR assays were used to define the *Bartonella* species in PCR+ participants.

Despite efforts to determine the *Bartonella* species, 13 of the 31 bacteremic participants were infected with an undetermined species, possibly due to very low bacterial numbers, or due to novel or known species for which the intergenic spacer primers lack sensitivity. This study confirmed that *B. henselae* was the most frequent species amplified from participant blood specimens (11/18). In addition, 6 participants, including two controls unaffected by psychosis, were infected with *Bartonella vinsonii* subsp. b*erkhoffii*, whose primary reservoir host is canids. Infection with *B. vinsonii* subsp. *berkhoffii* is considered an occupational risk for veterinary workers and others with extensive animal contact (39, 40). On a comparative medicine/One Health basis, a previous study involving cats examined following necropsy at the Animal Medical Center in New York documented an unexpectedly high prevalence of *B. vinsonii* subsp. *berkhoffii* DNA in endomyocarditis-left ventricular endocardial fibrosis cases compared to control cats with cardiomyopathy or histologically normal hearts (41). Findings in cats and humans in the New York City region justify future research efforts to define the mode(s) of transmission, potential reservoir(s), and medical importance of *B. vinsonii* subsp. *berkhoffii* in this location.

There was co-infection in three adults with psychosis, involving *B. quintana*, *B. alsatica*, and *B. rochalimae*. Although technically difficult to document with current testing modalities, *Bartonella* spp. co-infections have been previously reported, most often as a component of rigorous, complex research testing efforts (42–44). To our knowledge, *B. alsatica* has only been reported in a few human patients from Europe, comprising illnesses targeting the cardiovascular or lymphoid systems (45–48). Infection with *B. rochalimae* has been described in an American woman with fever, myalgia, and splenomegaly three weeks after multiple insect bites acquired during a trip in Peru (49). Additionally, *B*. *rochalimae* has been reported in association with endocarditis in a 22-year-old man who had unrepaired congenital ventricular septal defect, and in dogs in the United States (50, 51).

Similar to the North Carolina SCZ/SAD study, there was no significant difference in *Bartonella* spp. seroreactivity between the adults with psychosis and the controls. Based upon serology, *Bartonella* exposure was common among all study groups. As previously reported (28), there was low IFA sensitivity. For example, seven of 11 participants infected with *B. henselae* did not have detectable antibodies against this species, and five PCR+ participants were seronegative for all 5 antigens. This could be explained by anergy or antigenic variation among *Bartonella* strains resulting in false-negative IFA results in some participants (52). A significant increase of IgG antibodies against LPS but not against the *Bartonella* antigens tested in bacteremic adults could also account for anergy. Diminished antigen presentation was found in dogs experimentally infected with *B. vinsonii* subsp. *berkhoffii* (53), and IgG subclass deficiency has been reported in two women infected with *B. henselae* (54). Regardless of mechanism(s), these results suggest that serological tests are not clinically useful when attempting to assess the role of *Bartonella* spp. infections in participants with chronic psychiatric disorders. In addition to less-than-optimal sensitivity, cross-reactivity occurs across *Bartonella* spp. antigens, most prominently in endocarditis patients with extremely high IFA titers (55). Participants can also be co-infected with more than one *Bartonella* species, which further complicates interpretation of species cross-reactivity. In the context of specificity, cross-reactivity to other bacterial genera have been previously reported, but a recent publication failed to identify specific patterns of cross-reactivity across genera in occupationally at risk veterinary workers (10). As previously addressed (39, 40), serological and molecular results often did not agree in this study. None of the 6 participants who had a titer of ≥512 for at least one antigen was bacteremic, suggesting that anti-*Bartonella* antibodies could decrease the number of circulating bacteria below the level of molecular detection (56).

The frequency and medical importance of *Bartonella* spp. infections among family members is yet to be clarified. Although presumably an infrequent occurrence, perinatal transmission of *B*. *henselae* and *B*. *vinsonii* subsp. *berkhoffii* genotype II to twins in New York facilitated documentation of bacteremic durations that likely spanned a decade (52). In addition to the possibility of *in utero* infections, which clearly deserve increased research consideration, bacteremic infections with the same or different *Bartonella* species have been reported in multiple family members (52, 53). In the current study, two out of the four unrelated siblings of participants with psychosis were seroreactive to all five antigens. These two siblings were PCR-, whereas both participants with psychosis were bacteremic with either *Bartonella henselae* or an undetermined *Bartonella* species. The parent of one bacteremic participant was also included in this study; similar to her son unaffected by psychosis, she was seroreactive to all five antigens at titers ≥1:256 and PCR-. Three unrelated parents, without history of psychosis and whose offspring with psychosis was a child (1/3) or an adult (2/3), were PCR+. Interestingly, these two adults with psychosis and their parents were bacteremic; however, the *Bartonella* sp(p). infecting them was not determined using the techniques employed in this study. As long standing *Bartonella* sp(p). bacteremia is being increasingly confirmed with new, more sensitive diagnostic testing modalities, for example in blood donors and healthy veterinary workers (30, 34, 35, 54–56), documentation of asymptomatic infection in five controls in this study was an expected finding.

There were several limitations in this study. Due to the lack of aseptic technique and the manipulations of blood samples for prior testing purposes, culturing for *Bartonella* species was not performed. Thus, viable bacterial infection was not confirmed. The prevalence of *Bartonella* DNA in participants’ blood reported in this study was potentially underestimated because only a single blood specimen was tested, and enrichment blood culture was not performed (40). *Bartonella* is a highly fastidious bacterium that is difficult to document microbiologically in diagnostic specimens due to slow dividing times (approximately 22 hours), complex nutritional requirements, and intermittent bacteremia. To overcome these limitations in previous studies, our laboratory has required three aseptically collected blood and serum samples during a one-week period from each study participant, to increase the possibility of obtaining a PCR+ result (57). The presence of *Bartonella* DNA in blood was used to support infection; however, sequential testing would be necessary to confirm long-term bacteremia. In addition, the sample size for several groups was small; therefore statistical comparisons were limited to adults with psychosis compared to adult controls unaffected by psychosis. Finally, this study does not establish whether the presence of *Bartonella* spp. in the blood of adults with psychosis is a cause, a cofactor, or contributor to disease progression. Also, we cannot exclude the possibility of opportunistic infections, as *Bartonella* spp. infections have been associated with immune dysfunction (53, 54, 58). Our investigation had a limited infectious disease focus by design; testing for co-infection with other tickborne, vector-borne and non-vector-borne pathogens was not performed. As the *Bartonella* spp. test results were generated years after the original sample collection, antimicrobial therapy was not considered applicable.

On the basis of the North Carolina pilot study and the results of this study, there is justification for a large multi-center prospective study to determine if *Bartonella* spp. bacteremia is more prevalent in adults with psychosis compared to adults unaffected by psychosis and adults with other non-psychotic neurological disorders. Participants from different groups should match by age, sex, and socioeconomic status. Age of onset of symptomatology, as well as history of psychiatric hospitalizations (if any) and mental illness in the family, should be recorded. If such a future study supports an association between *Bartonella* spp. bacteremia and psychosis, *Bartonella*-targeted antimicrobial therapy trials could be initiated to determine if treatment improves or resolves psychotic behavior. Furthermore, broad infectious disease screening (including *Bartonella* spp.) should be considered in the setting of new onset neuropsychiatric disease, especially psychosis.

## Data availability statement

The original contributions presented in the study are included in the article/supplementary material. Further inquiries can be directed to the corresponding author.

## Ethics statement

The protocol for human sample collection (#7029) was approved by the New York State Psychiatric Institute Institutional Review Board. The studies were conducted in accordance with the local legislation and institutional requirements. Stored frozen blood and serum samples were de-identified prior to shipment to North Carolina State University for blinded serological and molecular testing purposes. Written informed consent for participation in this study was provided by the participant and, for those under age 18, by the participant's legal guardians/next of kin. Ethical approval was not required for the studies on animals in accordance with the local legislation and institutional requirements because only commercially available established cell lines were used.

## Author contributions

SD: Conceptualization, Resources, Writing – review & editing, Investigation. CR: Formal analysis, Investigation, Project administration, Writing – original draft, Writing – review & editing. RM: Conceptualization, Funding acquisition, Investigation, Methodology, Project administration, Writing – review & editing. EL: Writing – review & editing, Formal analysis. EK: Writing – review & editing, Investigation. CL: Investigation, Writing – review & editing. LM: Resources, Writing – review & editing. BF: Conceptualization, Resources, Supervision, Writing – review & editing. EB: Conceptualization, Funding acquisition, Supervision, Writing – original draft, Writing – review & editing.
